# Efficacy of Slow Sand Filtration Enriched with *Trichoderma atroviride* in the Control of *Fusarium oxysporum* in Soilless Cultivation Systems

**DOI:** 10.3390/pathogens15010091

**Published:** 2026-01-14

**Authors:** Pedro Matias, Luísa Coelho, Mário Reis

**Affiliations:** 1 Faculdade de Ciências e Tecnologia, Universidade do Algarve, Campus de Gambelas, 8005-139 Faro, Portugal; luisacoelho@greencolab.com (L.C.); mreis@ualg.pt (M.R.); 2 MED—Instituto Mediterrânico para a Agricultura, Ambiente e Desenvolvimento & CHANGE—Instituto para a Mudança Global e Sustentabilidade, Faculdade de Ciências e Tecnologia, Universidade do Algarve, Campus de Gambelas, 8005-139 Faro, Portugal; 3 CIMA/ARNET—Centro de Investigação Marinha e Ambiental/Rede de Investigação Aquática, Universidade do Algarve, Campus de Gambelas, 8005-139 Faro, Portugal; 4 Instituto Superior de Engenharia, Universidade do Algarve, Campus da Penha, 8005-139 Faro, Portugal; 5 GreenCoLab, Associação Oceano Verde, Universidade do Algarve, 8005-139 Faro, Portugal

**Keywords:** biological control, drainage, substrate cultivation, cucumber (*Cucumis sativus*), peat, plant disease control, antagonism, irrigation

## Abstract

On a planet intending to move toward carbon neutrality while ensuring food security, maximizing water and nutrient use efficiency in agriculture is essential. Soilless cultivation offers a promising solution for food production, yet in substrate-based systems, excess nutrient solution (drainage) is often discarded to maintain phytosanitary safety, resulting in considerable water and nutrient waste. Reusing this drainage requires disinfection to eliminate pathogens. Among available methods, slow sand filtration (SSF) is ecological, economical, and simple, showing strong biological control potential, though not always fully effective against *Fusarium oxysporum*. *Trichoderma atroviride*, an antagonistic fungus, may enhance SSF performance. Its antagonistic capacity was evaluated in vitro via direct confrontation assays and in vivo using a closed-loop soilless cucumber cultivation system with eight treatment combinations of SSF, *T. atroviride*, and *F. oxysporum*. SSF reduced *F. oxysporum* incidence by approximately 48%, *T. atroviride* in irrigation by 44%, and SSF enriched with *T. atroviride* reached 58% disease incidence reduction, though this increase was not statistically significant. These results confirm that both SSF and *T. atroviride* can partially suppress *F. oxysporum*, but further optimization is needed for consistent and complete pathogen control.

## 1. Introduction

Soilless cultivation is revolutionizing food production and holds significant potential to be a highly efficient and sustainable solution for meeting the food needs of a continuous growing global population [1,2,3,4,5,6]. Soilless cultivation involves the cultivation of plants, mostly vegetables or small fruits, without the use of soil, and can be performed in two primary ways: (i) Hydroponic cultivation—where plants are grown using only a complete nutrient solution, with no solid medium involved [7,8,9]; or (ii) Substrate cultivation—where plants are grown in a solid, porous medium that can be either organic (e.g., coconut fiber, peat) or inert (e.g., rockwool), irrigated with a nutrient solution [10,11,12]. The excess nutrient solution resulting from plant irrigation is referred to as drainage, and its management determines whether the soilless cultivation system is classified as open, closed, or semi-closed [2,13,14]. In an open system, the drainage is not reused to irrigate the same crop; instead, it is either discarded or used to irrigate a different crop [15,16]. In a closed system, the drainage is fully recycled for irrigating the same crop [13,17,18]. A semi-closed system typically operates like a closed system, but the drainage is only partially reused, to maintain the quality of the irrigation solution [2,10,15]. When crops are grown in a substrate, an open system is more common, with the drainage being discarded [2,18].

The use of open systems results in higher water consumption compared to closed systems, with usage being up to 42% higher [19,20,21,22,23]. In the Mediterranean region, which faces water scarcity [24,25,26] and where soilless cultivation is widely adopted, mostly in open systems, exploring strategies to reuse drainage is essential for reducing water demand. Additionally, open systems consume 15% to 80% more fertilizers than closed systems [22,27,28,29]. This results in unnecessary economic costs for producers, as well as environmental impacts related to greenhouse gas emissions, particularly nitrous oxide (N_2_O), which is closely associated with the production and use of nitrogen-based synthetic fertilizers [30,31,32]. However, these are not the only negative environmental impacts associated with open systems. Improper disposal of drainage can lead to soil and aquifer contamination [33]. Some countries regulate drainage disposal due to the environmental damage it may cause [34,35]. Reusing drainage is crucial for improving water and fertilizer use efficiency while avoiding or reducing various negative environmental impacts [18].

Reusing drainage for irrigation of the same crop in a closed system requires phytosanitary control to prevent the spread of phytopathogenic organisms. Without proper control, these organisms could rapidly disperse and infect a significant portion of the crop [36]. In a closed system, various phytopathogens, including oomycetes, fungi, bacteria, viruses, and nematodes, can be easily spread through [37,38,39,40,41], causing large economic losses [40,42]. Although it is not usually as problematic in soilless cultivation systems as *Pythium* spp. or *Phytophthora* spp., *Fusarium oxysporum* can cause significant losses if it occurs.

**Figure 1:**
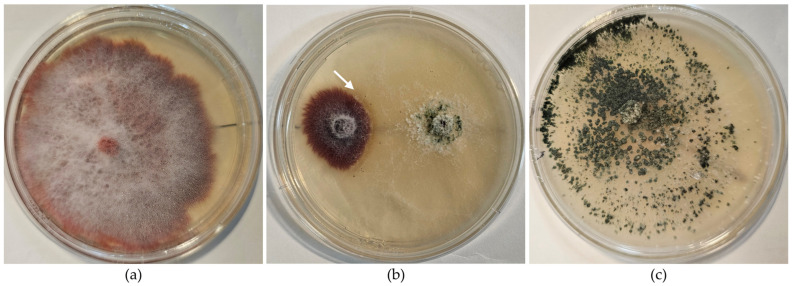
In vitro assessment of antagonistic capacity three days after inoculation: (**a**) *F. oxysporum*; (**b**) direct confrontation between *F. oxysporum* (left) and *T. atroviride* (right), showing the interaction zone (arrow); (**c**) *T. atroviride*.

*F. oxysporum* (Figure 1a) is a soil-borne, saprophyte fungus that is a significant plant pathogen, affecting a wide range of crops [43] by invading the vascular system of plants [44]. Being resistant to common control measures, it may cause substantial economic losses [45]. It can be particularly concerning in soilless systems due to its ability to spread rapidly through water [46], and persistence in growing media [47,48]. *F. oxysporum* infects plants through a multi-step process, starting with spore germination in response to root exudates (e.g., sugars, amino acids), forming hyphae [49,50,51]. Spore germination seems not to be host-specific and has been reported in tomato, sweet pepper, bean, barley, watermelon, rice, tobacco and cucumber [52,53]. The fungus penetrates the root system, usually through the root tips or small wounds, using specialized hyphae [54]. It then advances through the cortex of the root by secreting cell wall-degrading enzymes, such as pectinases and cellulases, which degrade plant cell walls, allowing to advance deeper into the root [55,56]. Once inside the xylem, *F. oxysporum* colonizes the vascular system, where it forms mycelium and produces spores that are carried through the plant’s sap flow, spreading the infection [44]. The fungus also secretes gels and toxins, clogging the xylem vessels and obstructing water and nutrient transport [44,57,58]. This results in wilting, yellowing, and stunted growth, despite the plant having access to sufficient water. As the infection progresses, the plant’s health declines, and *F. oxysporum* forms chlamydospores, highly resistant structures that enable the fungus to survive in soil or growing media for extended periods [59,60].

To prevent the spread of *F. oxysporum* and other pathogens, it is crucial to implement a disinfection system for the drainage before it is reintroduced for irrigation. Various water disinfection methods can be adopted, including physical treatments (e.g., heat, UV radiation, reverse osmosis), chemical treatments (e.g., iodine, chlorine, hydrogen peroxide, or ozone) [61,62], or biological methods (e.g., antagonistic microorganisms present in a filtration system) [63]. The slow sand filtration system (SSF) is an ecological, low-cost method that combines physical and biological actions, making it highly effective in controlling many pathogens in soilless cultivation systems [64]. Its low cost and ease of maintenance can be important advantages over other systems.

SSF, developed in 1804 by John Gibbs in Scotland, was initially used to purify water by physically removing suspended solids, before its effectiveness in controlling pathogenic microorganisms was known [65,66]. Adopted for public water treatment in 1829 and widely implemented after 1885, SSF proved highly effective during a cholera outbreak in Germany, which led to the recognition of its ability to control pathogenic microorganisms [66,67]. By the late 1980s, SSF was also applied in horticulture for disinfecting irrigation drainage, extending its utility beyond potable water treatment [64,68].

SSF is based on the slow flow of the nutrient solution through a granular medium, typically composed of fine sand or other materials with controlled grain size [64,68]. The low flow velocity and continuous retention of the solution above the filter medium promote the development of an active biological layer on the filter surface [68,69].

The biological layer and the filter bed operate through several mechanisms: (i) Physical filtration—suspended particles are retained within the filter bed, forming a layer of organic matter on its surface [64,69]; (ii) Biological layer formation—microbial community develops on the surface, decomposing the retained organic material and forming a biologically active layer known as the *schmutzdecke* [69,70]; (iii) Direct biological control—microorganisms within the *schmutzdecke* exert biocontrol over pathogenic organisms through various biological interactions as drainage flows through the filter [63,71]; and (iv) Chemical adsorption—dissolved compounds, nutrients, and pathogenic microorganisms are retained by the filter media through ion exchange, electrostatic attraction, and surface complexation processes as the drainage water percolates through the filter [72,73,74].

Pioneering authors in the use of this system for soilless cultivation recommend a filtration rate between 0.1 and 0.4 m^−3^ m^−2^ h, a filter bed height of 0.7–1.2 m, and surface cleaning every 4–12 weeks to maintain filtration efficiency [42,64,68,69,71]. This procedure scrapes the top 1–2 cm of sand, removes the old biofilm, allows regeneration of a new one, and may require replenishing part of the filter medium after repeated scrapes [69,70]. The sand used as filter media should present an effective grain size between 0.15 and 0.3 mm and a uniformity coefficient of up to 3, which may reach a maximum value of 5 [42]. This drainage disinfection system offers significant advantages over alternative methods: (i) It has low installation and maintenance costs, and requires minimal energy [41,70,75]; (ii) it is easy to operate [41]; (iii) it is not too much affected by water flow variations and does not require prior filtration [41,75]; and (iv) is highly effective against several pathogens. It has been shown to completely remove *Phytophthora* spp. [63,76], *Pythium* spp. [77,78], *Botrytis cinerea* [79], and others. However, it does not always fully eliminate *F. oxysporum* [63,76]. For large volumes of drainage to be treated, the filter requires considerable space, which also limits its mobility, as relocating involves transporting substantial amounts of sand [41,75].

Considering its limited effectiveness in eliminating *F. oxysporum*, enriching the microbial community within the filter could be a promising approach to enhance its overall efficiency. *Trichoderma atroviride* (Figure 1c) is a widely distributed filamentous soil fungus known for its strong ability to control various plant pathogens, including *Fusarium oxysporum* [80,81,82], *Rhizoctonia* spp. [18], *Sclerotinia* spp. [83], *Botrytis* spp. [84,85,86], *Pythium* spp. [87], and *Phytophthora* spp. [88].

*T. atroviride* employs multiple biocontrol mechanisms, both direct and indirect. Direct mechanisms include the following: (i) Mycoparasitism—parasitism of a host fungus, involving host detection, chemotropism, attachment, coiling, and host cell lysis [89,90,91], as observed against *F. oxysporum* [92]; (ii) Cell wall-degrading enzymes—production of extracellular enzymes such as chitinases, β-glucanases, and proteases that hydrolyze key fungal cell wall components [93,94,95]; (iii) Antibiotic synthesis—production of secondary metabolites, including peptaibols and volatile organic compounds, which disrupt *F. oxysporum* membranes and inhibit growth through antibiosis [82,96,97,98]; (iv) Competition for space and nutrients—rapid colonization of shared habitats, efficient carbohydrate metabolism, mobilization of essential elements, and siderophore-mediated iron sequestration restrict pathogen growth [99,100,101,102,103].

Indirect mechanisms include the induction of plant resistance in response to biotic stresses through induced systemic resistance. *T. atroviride* colonizes plant roots and produces signaling molecules that activate defense pathways, priming the plant to respond more rapidly and robustly to subsequent pathogen attacks without causing disease [104,105,106].

Due to its biocontrol mechanisms, which are effective against a wide range of plant pathogens, *T. atroviride* has been employed in the biological control of various diseases across multiple crops [107,108,109,110]. In soilless cultivation systems, *Trichoderma* spp. has been employed for biological control of *F. oxysporum* [111] and other plant pathogens [18,112,113,114].

We found that *T. atroviride* was able to enhance the efficacy of the slow filtration system in controlling *Rhizoctonia solani* [18]. Therefore, it was relevant to assess whether this effect also extends to *F. oxysporum*, since previous studies reported that SSF alone is not effective against this pathogen [63,76]. The objective of this study was thus to evaluate whether inoculating SSF with *T. atroviride* would improve its efficacy in controlling *F. oxysporum*.

## 2. Materials and Methods

### 2.1. Evaluation of Antagonistic Capacity—In Vitro

An in vitro assay was conducted to assess the antagonistic potential of the isolated strains of *T. atroviride* against *F. oxysporum*. *T. atroviride* was isolated from an agro-industrial waste compost obtained at the University of Algarve and identified using molecular methods [115]. The isolate showed 97% sequence coverage and 100% identity with the *T. atroviride* sequence MIAE00220. *F. oxysporum* was isolated from infected spinach (*Spinacia oleracea*) plants in parallel trials at the University of Algarve. The culture was initially identified macroscopically and microscopically (Labovert FS, Leitz, Germany) [116] and subsequently confirmed by molecular analysis. Both microorganisms were maintained on potato dextrose agar (PDA) (Biolife, Milan, Italy) at 24 °C (±1) in the dark.

The antagonistic potential was assessed using the direct confrontation method in Petri dishes containing PDA as the growth medium [117]. Two mycelial discs (6.5 mm in diameter), one of *T. atroviride* and one of *F. oxysporum*, were placed opposite each other in the same dish (Figure 1b). Additionally, the growth of each fungus, *F. oxysporum* (Figure 1a) and *T. atroviride* (Figure 1c), was evaluated individually under the same conditions. All plates were incubated at 24 °C for seven days to allow mycelial development.

The radial growth of each fungus, both in individual culture and in confrontation, was measured daily. The inhibition percentage (IP) was then calculated using the following formula:

(1) $$\mathrm{IP}\;=\;\frac{(\mathrm{Rc}\;-\;R1)}{\left(\mathrm{Rc}\right)}\;\times \;100$$

where the following definitions are used:

Rc—radius of the growth zone of the pathogen growing alone (mm);

R1—radius of the growth zone of the pathogen growing in the presence of the antagonist (mm).

### 2.2. Evaluation of Antagonistic Capacity—In Vivo

#### 2.2.1. Treatments and Experimental Design

Five consecutive in vivo trials were carried out using a closed soilless substrate cultivation system, in which organic cucumber seeds [*Cucumis sativus* L. ‘Marketer’, Semillas Fitó, Spain] were sown. This crop served as an indicator of disease presence due to its rapid and homogeneous germination, its susceptibility to *F. oxysporum*, and the easy identification of the symptoms. Each trial extended up to two weeks after seedling emergence, during which the plants served as indicators to assess the incidence and severity of *F. oxysporum*. The cultivation system was established in an unheated plastic-film greenhouse, equipped with natural ventilation through roof and side openings, located at the experimental field of the University of Algarve (Campus de Gambelas, Portugal).

The experimental design tested three factors: (i) the slow sand filter (F); (ii) the antagonist *Trichoderma atroviride* (T); (iii) the pathogen *Fusarium oxysporum* (P). The experimental treatments consisted of a combination of the presence (+) and absence (−) of each one of these factors, resulting in a total of 8 treatments: 1. F+T+P+; 2. F+T+P−; 3. F+T−P+; 4. F+T−P−; 5. F−T+P+; 6. F−T+P−; 7. F−T−P+; and 8. F−T−P−. Each of the treatments contained five pots that served as replications. In each pot were sown five cucumber seeds.

#### 2.2.2. Slow Sand Filter (SSF)

For treatments with the SSF (1. F+T+P+; 2. F+T+P−; 3. F+T−P+; 4. F+T−P−), the filter consisted of a vertical PVC column, 1 m in height and 10 cm in diameter, filled with 6 L of filtering material. This consisted of fine silica sand (0.85 m) (Maxmat, Porto, Portugal), layered above a 0.1 m layer of gravel at the base.

The particle size distribution of the filter media was determined by sieving [118]. The sand presented an effective particle size (d_10_) of 0.15 mm and a uniformity coefficient (UC) of 1.46, which falls within the range recommended by the pioneering authors in the development of slow sand filters (SSF) [42]. The gravel presented a d_10_ of 2.5 mm and a UC of 1.04. d_10_ corresponds to the particle diameter below which 10% of the particles are finer. The UC was calculated as the ratio between the sieve opening through which 60% (by weight) of the grains will pass and the effective grain size (UC = d_60_/d_10_).

For treatments without filtering (5. F−T+P+; 6. F−T+P−; 7. F−T−P+; 8. F−T−P−), the PVC columns were left empty.

#### 2.2.3. *Trichoderma atroviride* Growth and Inoculation

*T. atroviride* was introduced into the cultivation system as a conidial suspension at 10^6^ conidia mL^−1^ (T+ treatments: 1. F+T+P+; 2. F+T+P−; 5. F−T+P+; 6. F−T+P−), a standard concentration in biocontrol studies [119]. Prior to each trial, seven-day-old pure cultures of *T. atroviride* grown on PDA were washed with water to obtain the conidial suspension. Conidia concentrations were determined using a Neubauer chamber under a ×400 microscope (Labovert FS, Leitz, Germany). The required volume to achieve 10^6^ conidia mL^−1^ in the 6 L filter column was calculated and applied uniformly across all T+ treatments. For treatments with SSF (1. F+T+P+; 2. F+T+P−), the suspension was applied directly to the top sand layer of the filter, whereas in treatments without filtration (5. F−T+P+; 6. F−T+P−), it was added to the irrigation water. Inoculation was performed seven days before sowing.

#### 2.2.4. *Fusarium oxysporum* Growth and Inoculation

*F. oxysporum* was propagated in Petri dishes containing 40 mL of sterilized and neutralized blond peat, the same substrate used for plant growth. Each dish received five mycelial plugs (6.5 mm in diameter) and was incubated at 24 °C in the dark for seven days, until the peat was fully colonized by its mycelium. The colonized substrate was then transferred to pots matching the diameter of the Petri dishes, on the day of sowing. In P+ treatments (1. F+T+P+; 3. F+T−P+; 5. F−T+P+; 7. F−T−P+), these pots were placed on top of the PVC columns to receive the drainage from the irrigation channels.

#### 2.2.5. Cultivation System

**Figure 2:**
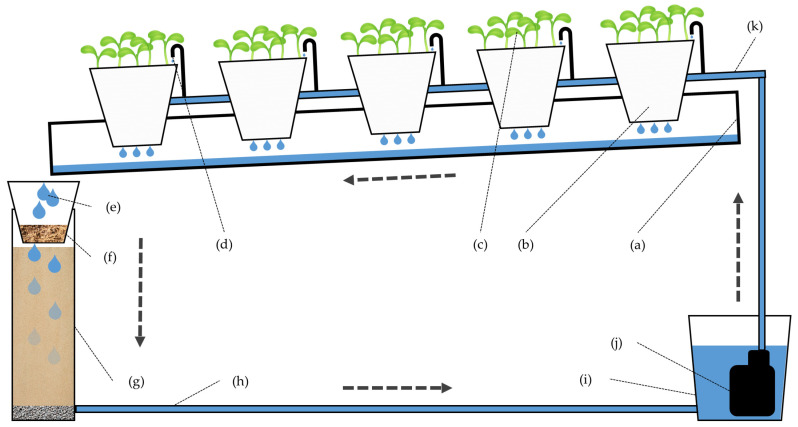
Scheme of the cultivation system used: (**a**) cultivation channel; (**b**) pot; (**c**) cucumber plants (*Cucumis sativus* L. ‘Marketer’); (**d**) microtube; (**e**) drainage collection; (**f**) substrate, with the pathogen only in the R+ treatments; (**g**) PVC pipe with sand in the F+ treatments and empty in the F− treatments; (**h**) (½” PE pipe) filtered drainage recover; (**i**) drainage collection tank (10 L); (**j**) submersible pump; (**k**) irrigation pipe. Black arrows indicate the direction of the irrigation water flow. This figure is identical to that published in a previous article by the same authors (DOI: https://doi.org/10.1016/j.cropro.2024.106917 [18]), who retain the intellectual property rights to the original version.

The cultivation system used was a prototype of a closed-loop substrate cultivation setup (Figure 2). Eight inclined gutters were installed, one for each treatment (Figure 2a), with five pots in each channel (Figure 2b). The pots were filled with blond peat, whose pH was adjusted to 7.0 by the addition of fine calcium carbonate. Five cucumber (*Cucumis sativus*) seeds were sown per pot, (Figure 2c).

Irrigation was supplied via a drip system (Figure 2d), and the drainage from each pot flowed into the channel, which collected the drainage from all pots and directed it into the PVC tube equipped either with a filter (1. F+T+P+; 2. F+T+P−; 3. F+T−P+; 4. F+T−P−) or left empty (5. F−T+P+; 6. F−T+P−; 7. F−T−P+; and 8. F−T−P−) (Figure 2e). In the P+ treatments (1. F+T+P+; 3. F+T−P+; 5. F−T+P+; 7. F−T−P+), an additional pot was placed on top of the PVC tube, filled with blond peat previously fully colonized in the laboratory by *F. oxysporum* (Figure 2f). The drainage passed through the filtering material (1. F+T+P+; 2. F+T+P−; 3. F+T−P+; 4. F+T−P−) or directly to an empty PVC tube (5. F−T+P+; 6. F−T+P−; 7. F−T−P+; and 8. F−T−P−) (Figure 2g): in all the filters, the drainage exited through an outlet (Figure 2h) connected to a 10 L reservoir (Figure 2i). From each drainage reservoir, a pump (Figure 2j) recirculated the solution back to the irrigation system, thus maintaining continuous irrigation (Figure 2k).

#### 2.2.6. Measurements

Disease assessment included evaluating disease severity (DS) and the percentage of infected plants (p), which were used to calculate disease incidence (DI), efficacy (E), consistency (C), the biological control index (BCI), and the control percentage (CP).

**Figure 3:**
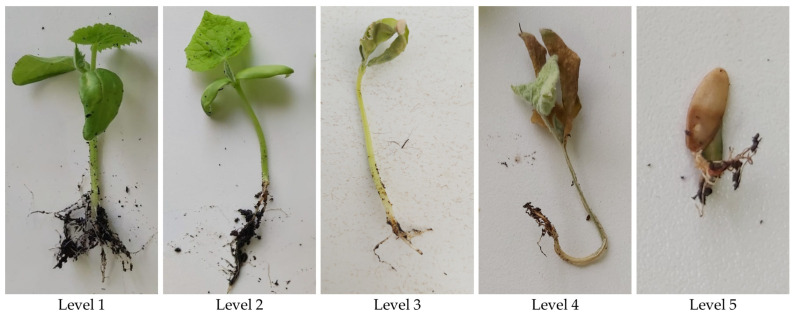
Visual scale of symptoms of *Fusarium oxysporum* on cucumber, to evaluate disease severity: Level 1—no symptoms; Level 2—mild lesions; Level 3—severe lesions; Level 4—post-emergence death; Level 5—pre-emergence death.

DS was rated for each plant using a 5-level visual symptom scale adapted from Baayen and van der Plas (1992) [120]: Lvl. 1—no symptoms; Lvl. 2—mild lesions; Lvl. 3—severe lesions; Lvl. 4—post-emergence death; Lvl. 5—pre-emergence death (Figure 3).

The presence or absence of disease on each plant was scored as 0 (healthy) or 1 (diseased). The number of infected plants per pot was counted to calculate the percentage of infected plants and, subsequently, the DI.

Efficacy (E) per pot was calculated as

(2) E=100−DI (%)

Consistency (C) for each treatment was expressed as the standard deviation of efficacy across replicates. The biological control index (BCI) per treatment was calculated following Byrne et al. (2005) [121]:

(3) $$BCI=\;\frac{E}{C}$$

The control percentage (CP) was calculated for each treatment using

(4) $$\mathrm{CP}=\left(1-\frac{{\mathrm{DI}}_{T}}{{\mathrm{DI}}_{T7}}\right)\;\times \;100\;(\%)$$

where DI_T7_ is the DI in plants inoculated only with *F. oxysporum* (7. F−T−P+), and DI_T_ is the DI for each other treatment.

#### 2.2.7. Statistical Analysis

Statistical analyses were performed using IBM^®^ SPSS^®^ Statistics 26. Disease severity (DS), assessed on a five-level scale, was analyzed using the non-parametric Kruskal–Wallis test due to the non-normal distribution of the data. Mean values were reported for descriptive purposes, and the percentage occurrence of each severity level was calculated. ANOVA followed by Duncan’s test was used to compare means of severity levels across treatments. Disease incidence (DI), efficacy (E), consistency (C), biological control index (BCI), and control percentage (CP) were analyzed using ANOVA and Duncan’s test. Pearson correlation coefficients between DS and DI were also calculated.

## 3. Results

### 3.1. Evaluation of Antagonistic Capacity—In Vitro

The antagonist *T. atroviride* displayed an average growth radius of 32 mm 72 h after its inoculation in Petri dishes. The pathogen *F. oxysporum* had a growth radius of 16 mm in the absence of *T. atroviride* and 11 mm in its presence, a reduction of 5 mm, resulting in a 28% inhibition rate.

### 3.2. Evaluation of Antagonistic Capacity—In Vivo

#### 3.2.1. Disease Severity

**Figure 4:**
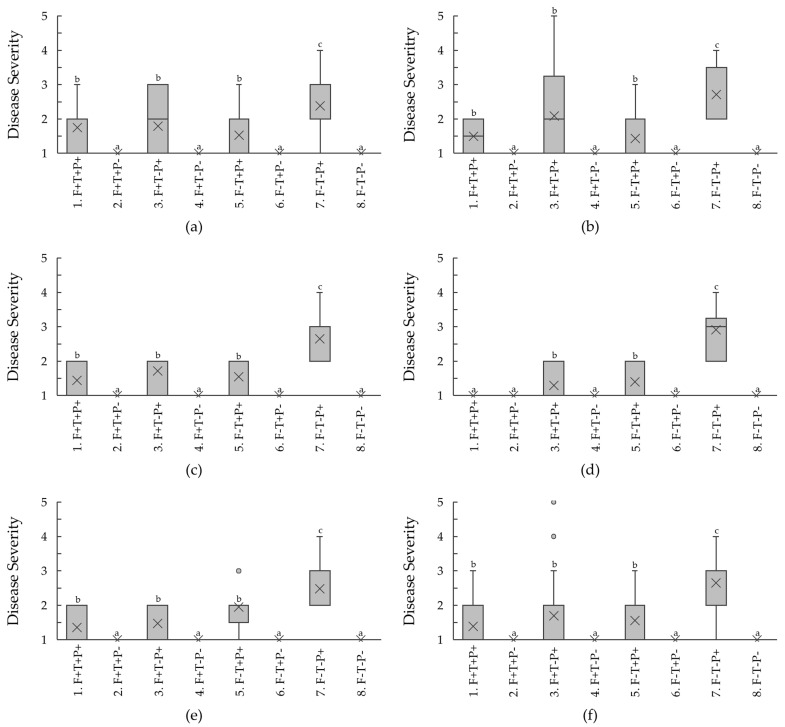
Distribution of disease severity levels per treatment, including its median (thicker lines), quartiles, interquartile range, and outliers: (**a**) first trial; (**b**) second trial; (**c**) third trial; (**d**), fourth trial; (**e**) fifth trial; (**f**) average of all trials. Equal letters indicate the absence of statistical difference, according to the non-parametric Kruskal–Wallis statistical test. F, filter; T, *Trichoderma atroviride*; P, *Fusarium oxysporum*; +/−, presence/absence.

The disease severity showed a clear pattern across all trials and in the overall average (Figure 4). In the treatments where the pathogen was not inoculated (2: F+T+P−; 4: F+T−P−; 6: F−T+P−; and 8: F−T−P−), the disease severity was consistently lower than in any other treatment (except only for treatment 1 in the fourth trial).

Treatments that included the pathogen and some form of control method, whether SSF alone (3: F+T−P+), SSF with *T. atroviride* (1: F+T+P+), or *T. atroviride* in the irrigation water (5: F−T+P+), exhibited similar levels of disease severity, but these were higher than in the treatments without the pathogen (2, 4, 6, and 8). The highest level of disease severity consistently occurred when the pathogen was present, and no control method was applied (7: F−T−P+). In this case, severity was always higher than in any other situation. Summarizing, we can group the treatments into three categories based on disease severity: (i) a minimal level, which includes all treatments without *F. oxysporum*, where the plants showed no disease symptoms; (ii) an intermediate level, including all treatments where *F. oxysporum* was present but a control method was applied; and (iii) the highest level, where the treatment contained *F. oxysporum* with no control method applied.

**Figure 5:**
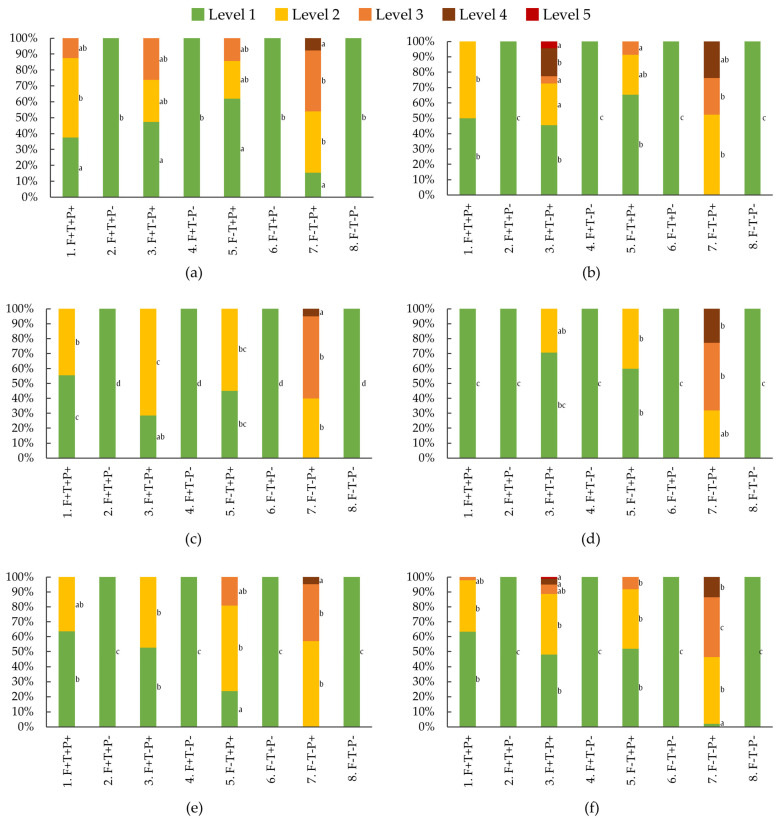
Average percentage of plants with each disease severity level (Lvl.), per treatment: (**a**) first trial; (**b**) second trial; (**c**) third trial; (**d**) fourth trial; (**e**) fifth trial; (**f**) average of the five trials. For each level of disease severity (Lvl.). Between treatments, the Lvl values with the same letter showed no statistical differences (*p* < 0.05), according to Duncan statistical Test. F, filter; T, *Trichoderma atroviride*; P, *Fusarium oxysporum*; +/−, presence/absence.

The occurrence of each severity level (%) shows that the lowest severity (Lvl. 1) consistently occurred in a higher percentage of plants in the treatments without the pathogen (2: F+T+P−; 4: F+T−P−; 6: F−T+P−; and 8: F−T−P−), except for treatment 1 in the fourth trial (Figure 5). In these treatments, 100% of the plants exhibited Lvl. 1 disease severity, meaning that none of the plants showed disease symptoms, as previously observed (Figure 4). All other treatments consistently showed lower percentages of Lvl. 1 disease severity. When *F. oxysporum* was present and no control method was applied (7: F−T−P+), plants almost never reached Lvl 1. This treatment consistently showed the lowest percentage of plants at Lvl 1 compared to all other treatments.

The remaining treatments (1: F+T+P+; 3: F+T−P+; and 5: F−T+P+) showed an intermediate percentage of plants at Lvl. 1. When these treatments showed severity levels above 1, it was almost always at level 2 (the second least severe level). When level 3 was observed, these treatments consistently had some of the lowest percentages of plants at that level. On average, in treatments with the pathogen and some form of control (1: F+T+P+; 3: F+T−P+; and 5: F−T+P+), between 37% and 52% of the plants showed disease symptoms. This percentage was significantly higher when no control method was used (7: F−T−P+), rising to 98%. This treatment also had, on average, a mortality rate of 13%, which was higher than any other treatment, although treatment 3 had a mortality rate of 5%.

#### 3.2.2. Disease Incidence

As observed in disease severity, disease incidence showed that the plants from the treatments without the pathogen (2: F+T+P−; 4: F+T−P−; 6: F−T+P−; and 8: F−T−P−) were never infected by *F. oxysporum* (Table 1). These treatments consistently exhibited an incidence of 0.0, which was statistically lower than the remaining treatments, except for treatment 1 in the fourth trial (Table 1). When *F. oxysporum* was present without any control method (7: F−T−P+), the disease incidence was higher than in any other treatment in the second and fourth trials, and overall mean, while in the remaining trials, it was similar to one of the treatments with a control method (1: F+T+P+; 3: F+T−P+; or 5: F−T+P+). In this treatment (7: F−T−P+), the disease incidence ranged from 83.3 to 100, reaching 100 in four out of five trials. Treatments with a control method (1: F+T+P+; 3: F+T−P+; and 5: F−T+P+) showed similar disease incidence in the first and second trials, as well as in the overall mean. In the third trial, disease incidence was higher in treatment 3 (F+T−P+) than in treatment 1 (F+T+P+); in the fourth and fifth trials, it was higher in treatment 5 (F−T+P+) than in treatments 1 (F+T+P+) and 3 (F+T−P+). On mean across the trials, these treatments (1: F+T+P+; 3: F+T−P+; and 5: F−T+P+) showed disease incidence values ranging from 39.2 to 52.4, which were 46% to 60% lower than in the absence of a control method (7: F−T−P+).

Just like disease severity, disease incidence appears to group the treatments into three distinct categories: (i) zero incidence group, with no disease—treatments without the pathogen (2: F+T+P−; 4: F+T−P−; 6: F−T+P−; and 8: F−T−P−); (ii) intermediate group, where the disease was partially present—treatments with the pathogen and some form of control method (1: F+T+P+; 3: F+T−P+; and 5: F−T+P+); and (iii) high incidence group—treatment with the pathogen and no control method (7: F−T−P+).

Disease incidence follows a pattern similar to that of disease severity. The strong and statistically significant correlation between these variables (Table 2) reinforces this similarity. This correlation indicates that as disease incidence increases, the disease severity also rises, and the reverse is true as well.

#### 3.2.3. Efficacy, Consistency, and Biological Control Index

Efficacy, consistency, and the biological control index once again demonstrate that there are differences between certain treatment groups (Table 3). Treatments without the pathogen (2: F+T+P−; 4: F+T−P−; 6: F−T+P−; and 8: F−T−P−) consistently achieved 100% efficacy, which was always higher than in all other treatments, except for treatment 1 in the fourth trial. In these treatments, consistency was always 0.0 because efficacy was 100% in all pots, and no biological control index was observed.

When *F. oxysporum* was present without any control method (7: F−T−P+), efficacy ranged from 0.0% to 16.7%, with 0.0% in four out of the five trials, almost always lower than all other treatments (except for treatments 1, 3, and 5 in the first trial, treatment 3 in the third trial, and treatment 5 in the fifth trial, where efficacy was similar). Whenever efficacy was 0.0%, consistency was also 0.0 because all pots were infected by the pathogen, leading to the absence of a biological control index. The average efficacy across all trials showed an efficacy of 3.3% in this treatment (7: F−T−P+), statistically lower than in all other treatments, with a consistency of 11.8, which resulted in a 0.71 biological control index. The remaining treatments, which included *F. oxysporum* and a control method (1: F+T+P+; 3: F+T−P+; and 5: F−T+P+), generally showed intermediate efficacy between the two situations previously discussed, with efficacy values that were almost always similar to each other. On average across all trials, these treatments (1: F+T+P+; 3: F+T−P+; and 5: F−T+P+) had efficacy ranging from 47.6% to 60.8%, statistically similar to each other, and higher to treatment 7 (F−T−P+) and lower than treatments 2 (F+T+P−), 4 (F+T−P−), 6 (F−T+P−), and 8 (F−T−P−). Consistency ranged from 37.6 to 41.3, resulting in a similar biological control index with values between 1.34 and 1.42, which was 47% to 50% higher than treatment 7.

#### 3.2.4. Control Percentage

The disease control percentage (Table 4) represents the level of control observed in each treatment and trial compared to the scenario where the pathogen was present without a control method (7: F−T−P+).

When no control method was applied and *F. oxysporum* was present (7: F−T−P+), the percentage of control was always 0.0%, statistically lower than all other treatments, except for treatments 1, 3, and 5 in the first trial, treatment 3 in the fifth trial, and treatment 5 in the fifth trial.

When the pathogen was absent (2: F+T+P−; 4: F+T−P−; 6: F−T+P−; and 8: F−T−P−), the control percentage was always 100%, higher than any other treatment, except for treatment 1 in the fourth trial. The remaining treatments, which included *F. oxysporum* and some control method (1: F+T+P+; 3: F+T−P+; and 5: F−T+P+), generally showed similar control percentages among themselves, with some variations in the third, fourth, and fifth trials. Whenever these treatments differed, treatment 1 consistently had the highest control percentage among the three treatments. On average, the percentage of control for these treatments ranged from 45.7% to 58.2%. This indicates that the tested control methods, SSF alone (3: F+T−P+), *T. atroviride* in the irrigation water (5: F−T+P+), or SSF combined with *T. atroviride* (1: F+T+P+), allowed the control of 45.7% to 58.2% of the pathogen.

## 4. Discussion

The in vitro antagonistic capacity results demonstrated that *T. atroviride* exhibits some control degree over *F. oxysporum*, achieving an inhibition rate of 28%. Other researchers have reported even higher inhibition rates, indicating that *T. atroviride* possesses some capacity to inhibit *F. oxysporum* [80,81,82].

In the in vivo trials, the disease did not develop when the pathogen was not inoculated, as evidenced by minimal disease severity, 0% disease incidence, and a control percentage and efficacy consistently at 100%, while the biological control index was not observed, as no biological control occurred. These results show that there was no cross-contamination between the treatments where the pathogen was inoculated and those where it was not.

When the pathogen was inoculated without any control method, *F. oxysporum* effectively spread throughout the cultivation system and successfully infected the plants, reaching an average disease incidence of 97%, the highest among all treatments (Table 1). This further shows that cucumber (*C. sativus*) is a crop affected by *F. oxysporum* [122,123,124]. With nearly all plants infected, disease symptoms were also most severe compared to other treatments, with a median severity level of 3 (Figure 4) and an average mortality rate of 13% (Figure 5). In 4 out of 5 trials, efficacy was zero, with an average of just 3%. The absence of healthy plants resulted in no BCI being observed in 4 out of 5 trials, where efficacy was 0% (Table 3). These results align with the findings of other researchers and demonstrate that when *F. oxysporum* is present, it can spread through water [125] and consequently in soilless cultivation systems, leading to plant infection [40,126]. In a closed substrate cultivation system (with drainage recirculation), *F. oxysporum* colonizes the substrate and infects the plants [48]. Therefore, for drainage reuse and in the presence of this pathogen, disinfecting the drainage is crucial to limit its spread within the cultivation system.

Three control methods were tested: (i) SSF; (ii) *T. atroviride* inoculated in the irrigation water; and (iii) *T. atroviride* inoculated in the sand of the filter. We found that these three methods showed similar effectiveness in controlling *F. oxysporum*, as evidenced by similar disease severity (Figure 4), disease incidence (Table 1), efficacy, BCI (Table 3), and control percentages (Table 4) across all three control methods. None of these methods were able to completely control *F. oxysporum*, as disease severity and incidence were always higher than when the pathogen was not inoculated. However, they were effective when compared to the absence of a control method, where disease severity and incidence were significantly higher and efficacy significantly lower. On average, those control methods achieved a control percentage between 46% and 58%, meaning that between 46% and 58% of the plants were not infected by the pathogen due to the respective control method. Although there were no significant differences between methods, the sand filter enhanced with *T. atroviride* showed, on average, the lowest incidence and the highest efficacy and control percentage.

Some researchers found that SSF was not able to reduce *Fusarium* spp. [76], others have found that SSF can partially reduce *F. oxysporum* without eliminating it [63,69], while others found that SSF can be highly effective in eliminating *F. oxysporum* and *Pythium* spp. from soilless tomato culture systems, achieving removal rates of 98% to 99.9% [127]. In this study, we observed that SSF achieved an average control percentage of 48%, meaning that nearly half of the plants were not infected, due to the presence of the SSF. Other researchers found that a different version of SSF, the horizontal-flow slow sand filter, effectively reduced viable *F. oxysporum* propagules by over 99.9% due to physical entrapment in the sand bed [36]. Certain design modifications to the SSF system, such as changing the filtration material, could potentially achieve 100% efficacy in controlling *F. oxysporum*, as grain size, pore diameter, and porosity impact filter performance [71]. Also, constructing a filter with a supernatant water layer of at least 21 cm above the sand layer can lead to a 99.9% reduction in *F. oxysporum* inoculum [64].

The use of *T. atroviride* in irrigation water has also proven to be a possible solution, achieving results similar to those achieved with SSF. Other researchers found that the application of *Trichoderma* spp. in irrigation water (via chemigation) significantly reduced the presence of white mold (*Sclerotinia sclerotiorum*) in tomatoes, while also increasing yield [128]. Other studies have shown that applying *Trichoderma harzianum* through irrigation water in soil-based tomato crops reduced the incidence of *F. oxysporum* by 13.3% to 52.5% [129]. In our observations, applying *T. atroviride* via irrigation resulted in a similar disease incidence of 52.4%, which is 44.3% lower than the incidence observed without any control method.

Although SSF alone (3: F+T−P+) and *T. atroviride* in the irrigation water (5: F−T+P+) showed similar results, enhancing SSF with the addition of *T. atroviride* (1: F+T+P+) did not significantly improve its ability to control *F. oxysporum* compared to SSF without *T. atroviride* (3: F+T−P+). Analyzing the effect of SSF with *T. atroviride* in controlling *R. solani*, we previously observed a 49% higher control rate compared to SSF without the antagonist, reducing the disease caused by *R. solani* by 75% to 100% [18]. For *F. oxysporum* control, the results were similar between SSF with *T. atroviride* and SSF alone, and the SSF with *T. atroviride* reducing the disease caused by *F. oxysporum* by an average of 58%. Other researchers, although they observed lower disease incidence values against *F. oxysporum* compared to ours, found no significant differences between SSF alone (5.8%) and SSF with *Trichoderma* spp. (4.3%) [130]. Similarly, other studies also found no differences in *F. oxysporum* disease incidence between SSF (3.7%) and SSF enhanced with *Trichoderma* spp. (4.0%) [131].

This lack of additional control provided by the filter inoculated with *T. atroviride* (1. F+T+P+) compared to the presence of the filter alone (3. F+T−P+) or *T. atroviride* applied in the irrigation water (5. F-T+P+) may be attributed to several factors. Although *T. atroviride* exhibits antagonistic activity against *F. oxysporum*, it is not able to eliminate the pathogen, which could explain why the results were not superior to those obtained with application solely through the irrigation water. In our in vitro tests, *T. atroviride* achieved an inhibition rate of 28% against *F. oxysporum*, whereas *T. harzianum* has been reported to reach 87% [132] to 92% [133]. Other species, such as *T. asperellum* [134,135] and *T. koningii* [136], have also shown promising results in controlling *F. oxysporum*. Furthermore, the filter itself could potentially benefit from prolonged colonization by *T. atroviride*, as its antagonistic capacity increases with the duration of inoculation [137,138]. Modifications in the filter design, the selection of the antagonist species, and the inoculation period may ultimately enhance the efficacy of the inoculated filter against this pathogen.

## 5. Conclusions

Slow sand filtration (SSF) enriched with *Trichoderma atroviride* showed moderate efficacy in controlling *Fusarium oxysporum* in substrate cultivation systems. Although none of the tested control methods completely suppressed the pathogen, they all significantly reduced disease severity and incidence compared to the control.

The SSF alone achieved an efficacy of 49.5%, while the application of *T. atroviride* through irrigation had an efficacy of about 47.6%. *T. atroviride* with SSF resulted in an efficacy of 60.8%, although it was not statistically higher to the previous cases. Compared to the pathogenic treatment without any control method, the control percentage of SSF alone and *T. atroviride* in water ranged from 47.5% to 45.7%, respectively. When SSF was inoculated with *T. atroviride*, the control reached 58.2%, although the differences were not statistically significant.

Overall, SSF and *T. atroviride* applications both demonstrated partial control of *F. oxysporum*. However, to achieve higher efficacy, further optimization of filter design, filtration media, and inoculation strategies is required.

## Figures and Tables

**Table 1:** Disease incidence, caused by *Fusarium oxysporum*, in five trials (First–Fifth) and the mean of the five trials (Mean). The values presented are the mean ± standard error.

	Trial					
Treatment *	First	Second	Third	Fourth	Fifth	Mean
1. F+T+P+	65.0 ± 21.8 b	53.7 ± 14.7 b	40.0 ± 24.5 b	0 ± 0 a	37.3 ± 12.4 b	39.2 ± 8.3 b
2. F+T+P−	0 ± 0 a	0 ± 0 a	0 ± 0 a	0 ± 0 a	0 ± 0 a	0 ± 0 a
3. F+T−P+	50.0 ± 22.4 b	60.0 ± 19.0 b	75 ± 11.6 cd	20 ± 20 ab	47.7 ± 9.7 b	50.5 ± 7.9 b
4. F+T−P−	0 ± 0 a	0 ± 0 a	0 ± 0 a	0 ± 0 a	0 ± 0 a	0 ± 0 a
5. F−T+P+	48.0 ± 22.4 b	36.0 ± 13.7 b	58 ± 13.3 bc	40 ± 16.7 b	80 ± 15.5 c	52.4 ± 7.5 b
6. F−T+P−	0 ± 0 a	0 ± 0 a	0 ± 0 a	0 ± 0 a	0 ± 0 a	0 ± 0 a
7. F−T−P+	83.3 ± 10.5 b	100 ± 0 c	100 ± 0 d	100 ± 0 c	100 ± 0 c	96.7 ± 2.4 c
8. F−T−P−	0 ± 0 a	0 ± 0 a	0 ± 0 a	0 ± 0 a	0 ± 0 a	0 ± 0 a
F	6.029	14.911	14.134	14.790	26.459	53.419
Sig.	<0.001	<0.001	<0.001	<0.001	<0.001	<0.001

* F, sand filter; T, *Trichoderma atroviride*; P, *Fusarium oxysporum*; +/−, presence/absence. In each column, mean values with equal letters do not differ significantly, according to Duncan’s test (*p* = 0.05). The F-statistic (F) and significance (sig.) from the ANOVA statistical analysis are also presented for each trial (First–Fifth) and for the mean of the five trials (Mean).

**Table 2:** Correlation coefficient and significance between disease severity and disease incidence in five trials (First–Fifth) and the mean of the five trials (Mean).

	Trial					
	First	Second	Third	Fourth	Fifth	Mean
Correlation coefficient	0.975	0.897	0.948	0.916	0.938	0.924
Significance	<0.001	<0.001	<0.001	<0.001	<0.001	<0.001

**Table 3:** Efficacy (E), consistency (C) and biological control index (BCI) in five trials (First–Fifth) and the mean of the five trials (Mean).

	**Trial**								
	**First**			**Second**			**Third**		
Treatment *	**E (%)**	**C**	**BCI**	**E (%)**	**C**	**BCI**	**E (%)**	**C**	**BCI**
1. F+T+P+	35.0 a	48.7	0.72	46.3 b	32.9	1.41	60 c	54.8	1.10
2. F+T+P−	100 b	0.0	-	100 c	0.0	-	100 d	0.0	-
3. F+T−P+	50.0 a	50.0	1.0	40.0 b	42.4	0.94	25 ab	26.0	0.96
4. F+T−P−	100 b	0.0	-	100 c	0.0	-	100 d	0.0	-
5. F−T+P+	52.0 a	50.2	1.04	64.0 b	30.7	2.08	42 bc	29.7	1.41
6. F−T+P−	100 b	0.0	-	100 c	0.0	-	100 d	0.0	-
7. F−T−P+	16.7 a	23.6	0.71	0.0 a	0.0	-	0.0 a	0.0	-
8. F−T−P−	100 b	0.0	-	100 c	0.0	-	100 d	0.0	-
F	6.029	-	-	14.911	-	-	14.134	-	-
Sig.	<0.001	-	-	<0.001	-	-	<0.001	-	-
	**Trial**								
	**Fourth**			**Fifth**			**Mean**		
Treatment *	**E (%)**	**C**	**BCI**	**E (%)**	**C**	**BCI**	**E (%)**	**C**	**BCI †**
1. F+T+P+	100 c	0.0	-	62.7 b	27.7	2.26	60.8 b	41.3	1.37 ± 0.3 b
2. F+T+P−	100 c	0.0	-	100 c	0	-	100 c	0	-
3. F+T−P+	80.0 bc	44.7	1.79	52.3 b	21.7	2.42	49.5 b	39.7	1.42 ± 0.3 b
4. F+T−P−	100 c	0.0	-	100 c	0	-	100 c	0	-
5. F−T+P+	60.0 b	37.4	1.60	20.0 a	34.6	0.58	47.6 b	37.6	1.34 ± 0.3 b
6. F−T+P−	100 c	0.0	-	100 c	0	-	100 c	0	-
7. F−T−P+	0.0 a	0.0	-	0.0 a	0	-	3.3 a	11.8	0.71 ± −a
8. F−T−P−	100 c	0.0	-	100 c	0	-	100 c	0	-
F	14.790	-	-	26.459	-	-	53.419	-	11.149
Sig.	<0.001	-	-	<0.001	-	-	<0.001	-	<0.001

* F, sand filter; T, *Trichoderma atroviride*; P, *Fusarium oxysporum*; +/−, presence/absence. In each column, mean values with equal letters do not differ significantly, according to Duncan’s test (*p* = 0.05). The F-statistic (F) and significance (sig.) from the ANOVA statistical analysis are also presented for each trial (First–Fifth) and for the mean of the five trials (Mean). † Values presented are mean ± standard error.

**Table 4:** Control percentage (CP) in five trials (First–Fifth) and the mean of the five trials (Mean). Values presented are the mean ± standard error.

	Trial					
Treatment *	First	Second	Third	Fourth	Fifth	Mean
1. F+T+P+	22.0 ± 26.2 a	46.3 ± 14.7 b	60 ± 24.5 c	100 ± 0 c	62.7 ± 12.4 b	58.2 ± 9.06 b
2. F+T+P−	100 ± 0 b	100 ± 0 c	100 ± 0 d	100 ± 0 c	100 ± 0 c	100 ± 0 c
3. F+T−P+	40 ± 26.8 a	40 ± 19.0 b	25 ± 11.6 ab	80 ± 20 bc	52.3 ± 9.68 b	47.5 ± 8.43 b
4. F+T−P−	100 ± 0 b	100 ± 0 c	100 ± 0 d	100 ± 0 c	100 ± 0 c	100 ± 0 c
5. F−T+P+	42.4 ± 26.9 a	64 ± 13.7 b	42 ± 13.3 bc	60 ± 16.7 b	20 ± 15.5 a	45.7 ± 7.99 b
6. F−T+P−	100 ± 0 b	100 ± 0 c	100 ± 0 d	100 ± 0 c	100 ± 0 c	100 ± 0 c
7. F−T−P+	0 ± 12.6 a	0 ± 0 a	0 ± 0 a	0 ± 0 a	0 ± 0 a	0 ± 2.31 a
8. F−T−P−	100 ± 0 b	100 ± 0 c	100 ± 0 d	100 ± 0 c	100 ± 0 c	100 ± 0 c
F	6.029	14.911	14.134	14.790	26.459	50.009
Sig.	<0.001	<0.001	<0.001	<0.001	<0.001	<0.001

* F, sand filter; T, *Trichoderma atroviride*; P, *Fusarium oxysporum*; +/−, presence/absence. In each column, mean values with equal letters do not differ significantly, according to Duncan’s test (*p* = 0.05). The F-statistic (F) and significance (sig.) from the ANOVA statistical analysis are also presented for each trial (First–Fifth) and for the mean the five trials (Mean).

## Data Availability

The data will be made available upon request to the authors.

## Abbreviations

The following abbreviations are used in this manuscript:

SSF	Slow sand filtration
F	Sand filter
T	*Trichoderma atroviride*
P	*Fusarium oxysporum*
PVC	Polyvinyl Chloride
UC	Uniformity coefficient
PDA	Potato dextrose agar
IP	Inhibition percentage
DS	Disease severity
p	Percentage of infected plants
DI	Disease incidence
E	Efficacy
C	Consistency
BCI	Biological control index
CP	Control percentage
